# Establishment of a novel scoring model for mortality risk prediction in HIV-infected patients with cryptococcal meningitis

**DOI:** 10.1186/s12879-021-06417-9

**Published:** 2021-08-10

**Authors:** Ting Zhao, Xiao-Lei Xu, Jing-Min Nie, Xiao-Hong Chen, Zhong-Sheng Jiang, Shui-Qing Liu, Tong-Tong Yang, Xuan Yang, Feng Sun, Yan-Qiu Lu, Vijay Harypursat, Yao-Kai Chen

**Affiliations:** 1 Division of Infectious Diseases, Chongqing Public Health Medical Center, 109 Baoyu Road, Shapingba, Chongqing, 400036 China; 2 Department of Infectious Diseases, The Fourth Affiliated Hospital of Harbin Medical University, Harbin, Heilongjiang province China; 3 Division of Infectious Diseases, Liuzhou People’s Hospital, Liuzhou, Guangxi province China; 4 Department of Infectious Diseases, Guiyang Public Health Clinical Center, Guiyang, Guizhou province China; 5 Department of Infectious Disease, Public Health Clinical Center of Chengdu, Chengdu, Sichuan province China; 6 Department of Infectious Diseases, Sixth People’s Hospital of Zhengzhou, Zhengzhou, Henan province China

**Keywords:** HIV, Cryptococcal meningitis, Risk factors, Mortality, Scoring model

## Abstract

**Background:**

Cryptococcal meningitis (CM) remains a leading cause of death in HIV-infected patients, despite advances in CM diagnostic and therapeutic strategies. This study was performed with the aim to develop and validate a novel scoring model to predict mortality risk in HIV-infected patients with CM (HIV/CM).

**Methods:**

Data on HIV/CM inpatients were obtained from a Multicenter Cohort study in China. Independent risk factors associated with mortality were identified based on data from 2013 to 2017, and a novel scoring model for mortality risk prediction was established. The bootstrapping statistical method was used for internal validation. External validation was performed using data from 2018 to 2020.

**Results:**

We found that six predictors, including age, stiff neck, impaired consciousness, intracranial pressure, CD4^+^ T-cell count, and urea levels, were associated with poor prognosis in HIV/CM patients. The novel scoring model could effectively identify HIV/CM patients at high risk of death on admission (area under curve 0.876; *p*<0.001). When the cut-off value of 5.5 points or more was applied, the sensitivity and specificity was 74.1 and 83.8%, respectively. Our scoring model showed a good discriminatory ability, with an area under the curve of 0.879 for internal validation via bootstrapping, and an area under the curve of 0.886 for external validation.

**Conclusions:**

Our developed scoring model of six variables is simple, convenient, and accurate for screening high-risk patients with HIV/CM, which may be a useful tool for physicians to assess prognosis in HIV/CM inpatients.

## Background

Cryptococcal meningitis (CM), caused by infection with the fungal pathogen *Cryptococcus neoformans*, is a leading opportunistic infection in humans, and a common cause of meningitis in adults living with Human Immunodeficiency Virus (HIV) [1–4]. Despite advances in CM diagnostic and therapeutic strategies, early mortality from CM in the developing world remains high during the therapeutic antifungal induction and consolidation phases [5]. It has been proposed that the reason for this is that early diagnosis of CM has always been clinically challenging [6, 7].

Previous studies have focused on identifying key predictors of early mortality in patients who died within 2 to 10 weeks of initiating treatment. Many potential risk factors, like age, impaired consciousness, and CD4^+^ T-cell counts have been evaluated for their potential to predict mortality in HIV infected patients with CM (HIV/CM patients) [8–10]. Also, high intracranial pressure (ICP), blood neutrophil counts, cerebrospinal fluid (CSF) leukocyte counts, and CSF glucose levels have been evaluated in several studies as potential predictors of risk for development of CM [3, 5, 8]. Although these clinical features and laboratory findings have been rigorously studied and discussed for utility as prognostic tools in HIV/CM patients, thus far, the validity of the relationship between these factors and CM mortality for this group of patients remains disappointingly unclear. Up until now, there has not been the successful development of a multi-parameter scoring system to prognostically evaluate the potential for mortality in HIV/CM patients [11].

We therefore comprehensively considered clinical symptoms and signs of CM, characteristics of cerebrospinal fluid changes in CM, and prognosis in CM, and have developed a scoring model to accurately clinically classify and stage CM for attending clinicians working in primary hospital settings. Using the proposed scoring model, clinicians may accurately predict clinical outcomes in HIV/CM patients at admission from assessment of basic clinical parameters, thus facilitating expeditious and appropriate therapeutic interventions [12].

## Methods

### Study design and patient population

This retrospective multi-center cohort study included HIV-infected patients diagnosed with CM from 2013 to 2020 at the following 10 Chinese medical centers: Chongqing Public Health Medical Center, The First Hospital of Changsha, Liuzhou General Hospital, Guiyang Public Health Clinical Center, Public Health Clinical Center of Chengdu, Yunnan Provincial Infectious Disease Hospital, The Fourth Affiliated Hospital of Harbin Medical University, Guangxi Longtan Hospital, The First Affiliated Hospital of Zhejiang University, and The Sixth People’s Hospital of Zhengzhou. Data from all 10 centers from 2013 to 2017 were used to develop the derivation cohort. The validation cohort was based on data obtained from 6 of the 10 mentioned medical centers above between 2018 and 2020.

### Study parameters, study endpoint, and outcomes

The patients’ clinical data, including age, headache, maximum body temperature, stiff neck, impaired consciousness, weight loss, neutrophil count, total bilirubin (TBIL), hemoglobin (HGB), platelet (PLT), urea, creatinine, CD4^+^ T-cell count, HIV RNA viral load, ICP, CSF glucose, clinical outcome, and other clinical data were analyzed. The observation starting point was HIV-associated CM diagnosis after admission to hospital. The observation endpoint was patient death, or survival at 28 days after HIV/CM diagnosis. The survival rate during the 28 days post-diagnosis was calculated as the endpoint event.

### Definitions

Confirmation of HIV infection was established by positivity of the HIV antibody test via Western Blot, and a positive HIV RNA polymerase chain reaction [13]. The diagnosis of CM was based on the identification of *Cryptococcus neoformans* in CSF via India ink staining, CSF culture or CSF cryptococcal antigen assay [14, 15].

### Statistical analysis

Categorical variables were compared using the Chi-squared test or Fisher’s Exact test, and continuous variables were compared using Student’s t-test or the Mann-Whitney U-test. The Kolmogorov–Smirnov test was used to assess the normality of the sample data distribution. Data are presented as mean ± standard deviation, or median (interquartile range) for continuous variables, and as frequency and percentage for categorical variables.

In the derivative cohort, the relationship between baseline demographic characteristics, clinical characteristics, and laboratory data and all-cause mortality (as measured at day 28) were examined by univariate analyses. Variables with *p* values less than 0.1 in the above steps were further included in the multivariate logistic regression analysis. Multivariate analyses were performed to identify predicted risk factors for death. From the logistic regression model, coefficient scores were extracted and assigned for each factor with their respective β coefficients. To make the scores approach an integer or relative integer, and in order to be intuitive for the user, we arbitrarily assigned the regression coefficient of ICP (0.788) as equivalent to one risk point (risk score = 1), and all other β coefficients were then divided by 0.788 to determine the score of coefficient for each risk factor (rounded to the nearest number in units of 0.5). For example, the coefficient of 1.505 associated with CD4^+^ T-cell counts ≤50 cells/μL was divided by 0.788, and the answer thus obtained (i.e., 1.910) was rounded-off to 2.0. A specific risk score is derived by a calculation of each coefficient score multiplied by each point score. The risk score for an individual was obtained by summation of the scores for each of the risk factors.

To verify the validity of the scoring model, a receiver operating characteristic curve (ROC) was plotted and an area under the curve (AUC) was calculated [16]. The performance of the new scoring model was internally validated using the bootstrapping statistical method, with 1000 replicates. External validation was also performed to further evaluate the discriminative ability of the newly developed model to predict the risk of HIV/CM mortality in the validation cohort.

Statistical analyses were performed using the SPSS software package for Windows (Version 23; IBM-SPSS, Inc., Chicago, IL, USA) and R statistical software (Version 4.0.3, The R Foundation of Statistical Computing, Vienna, Austria). For all tests, a *p*-value < 0.05 was considered to be statistically significant.

## Results

**Fig. 1:**
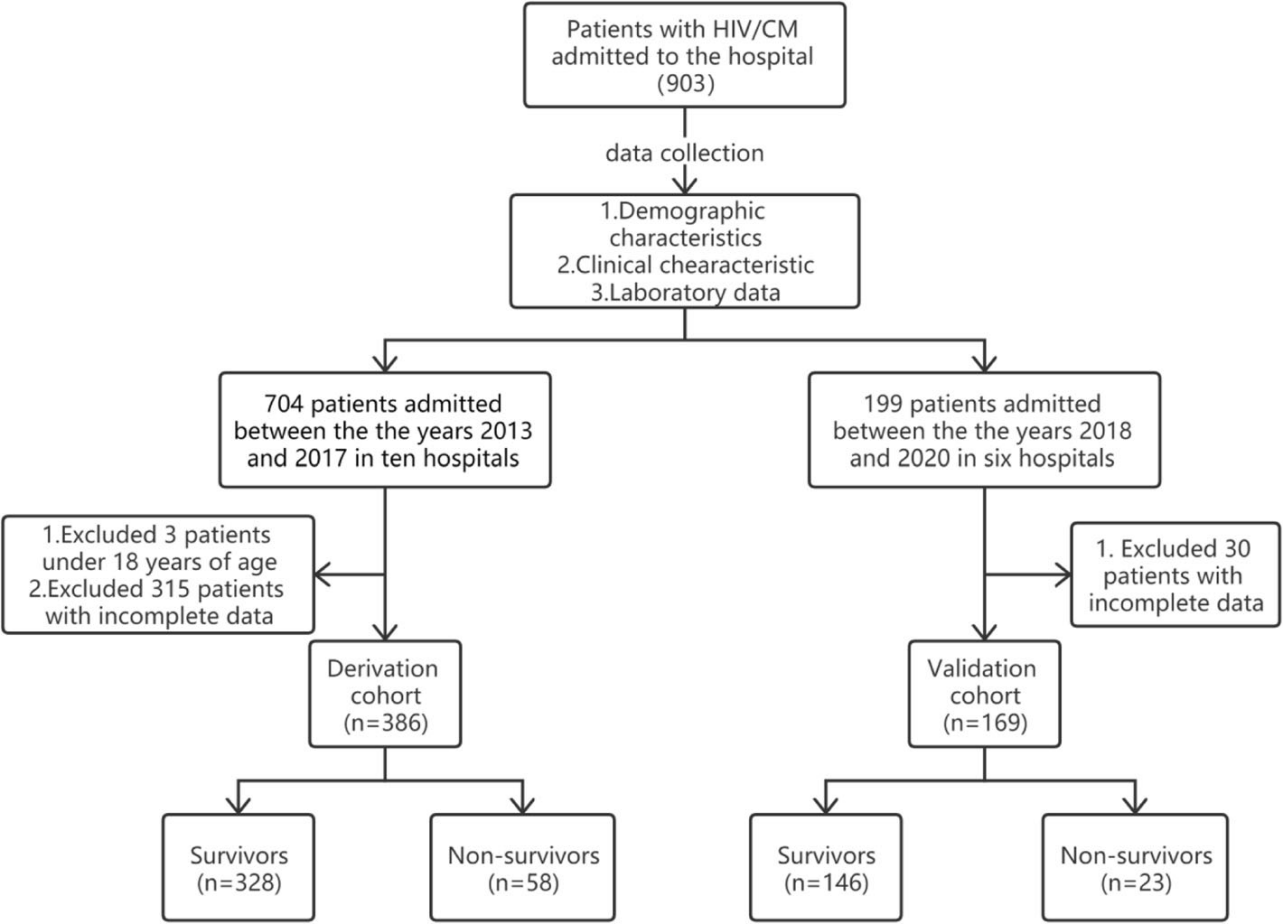
Flow chart of inclusion and exclusion of study participants

A total of 903 HIV/CM patients from 10 participating hospitals were screened in this study. Of these, 348 patients were excluded due to data loss (with missing test results, laboratory data, or without complete demographic characteristics), and three patients were excluded for being under 18 years of age. As a result, 386 patients were assigned to the derivation cohort, of which 328 survived and 58 died. The all-cause mortality rate was 15.0%. Of the 169 patients in the validation cohort, 23 patients succumbed to CM, with an all-cause mortality rate of 13.6%. A flow chart of our study population selection and study process is shown in Fig. 1.

### Comparison of clinical biochemical parameters and baseline characteristics in the derivation cohort

To identify risk factors for mortality in CM, we evaluated the relationship between deaths and the demographic, clinical, comorbidity and complications, and laboratory characteristics of the screened individuals by performing univariate analyses on these data. The examined characteristics included demographic characteristics, clinical characteristics, comorbidity and complications, and laboratory data, as summarized in Table 1. By comparing the 29 measured clinical factors between the survivors and non-survivors, six factors including impaired consciousness, stiff neck, age, TBIL, ICP, and PLT levels were found to have a significant influence on mortality risk (*p <* 0.0001). In addition, HIV RNA viral load, CSF glucose, and urea levels were associated significantly with patient mortality (*p <* 0.05). However, the analysis of other clinical and laboratory data, comorbidity and complications, including presence of headache, weight loss, neutrophil count, creatinine, CD4^+^ T-cell count, sex, ART at hospital admission, high body temperature, seizures, time from symptom onset to hospital admission, whether in association with bacterial pneumonia, pneumocystis pneumonia, tuberculosis, cytomegalovirus infection, diabetes, liver cirrhosis, and cerebral infarction did not differ statistically between the survival and non-survival populations (*p* > 0.05, Table 1).

**Table 1:** Univariate analyses of baseline characteristics of HIV/CM inpatients in the derivative cohort

Variable	Survivors	Non-survivors	***p-***value
	(***n*** = 328)	(***n*** = 58)	
Demographic characteristics			
Age (years, IQR)	43(34,52)	54(44.8,65)	< 0.0001
Male sex, n (%)	263 (80.2%)	40 (69.0%)	0.055
Comorbidity and Complications			
Cerebral infarction, n (%)	14 (4.3%)	5 (8.6%)	0.182^#^
Liver cirrhosis, n (%)	5 (1.5%)	2 (3.4%)	0.284^#^
Diabetes, n (%)	17 (5.2%)	5 (8.6%)	0.351^#^
Tuberculosis, n (%)	47 (14.3%)	12 (20.7%)	0.215
Pneumocystis pneumonia, n (%)	30 (9.1%)	6 (10.3%)	0.772
Bacterial pneumonia, n (%)	48 (14.6%)	13 (22.4%)	0.134
Cytomegalovirus infection	34 (10.4%)	7 (12.1%)	0.698
Clinical characteristic			
Time from symptom onset to hospital admission (month, IQR)	0.7(0.3,1.0)	1(0.3,2.0)	0.287
Maximum body temperature (°C, IQR)	37.8 (36.7,39.0)	36.6 (36.7,38.3)	0.213
Stiff neck, n (%)	24 (7.3%)	17 (29.3%)	< 0.0001
Impaired consciousness, n (%)	28 (8.5%)	22 (37.9%)	< 0.0001
Headache, n (%)	243 (74.1%)	45(77.6%)	0.572
Seizures, n (%)	25 (7.6%)	7 (12.1%)	0.298^#^
Weight loss, n (%)	125 (38.1%)	29 (50.0%)	0.088
Laboratory data			
CSF glucose (mmol/L, IQR)	2.54 (1.9,3.2)	2.25 (1.2,3.1)	0.045
Neutrophil count (10^9^/L, IQR)	3.4 (2.3,5.4)	3.8 (2.1,6.3)	0.419
TBIL (μmol/L, IQR)	9.2 (7.0,13.6)	13.5 (9.0,19.7)	< 0.0001
HGB (g/L, IQR)	115.0 (98.0,130.0)	108.0 (94.3126.0)	0.235
PLT (× 10^6 /^L, IQR)	186 (126.0,248.8)	120 (82,202)	< 0.0001
Urea (mmol/L, IQR)	3.8 (3.0,5.5)	4.9 (3.7,6.8)	0.001
Creatinine (μmol/L, IQR)	59.1 (49.0,71.2)	60.3 (49.1,75.6)	0.787
White blood cell count (10^9^/L, IQR)	4.6 (3.4,6.5)	4.7(3.1,7.2)	0.939
CD4^+^ T-cell count (cells/μL, IQR)	32.0 (13.0,77.3)	29.0 (12.8,48.0)	0.095
HIV RNA (log10 copies/mL, IQR)	5.1 (3.9,5.7)	5.4 (4.9,5.9)	0.009
ICP (mm H_2_O, IQR)	215.0 (160.0,328.0)	360.0 (247.0,400.0)	< 0.0001
ART at hospital admission, n (%)	198 (51.3%)	27(46.6%)	0.500

*Abbreviations*: *CSF* cerebrospinal fluid, *HGB* hemoglobin, *ICP* intracranial pressure, *TBIL* total bilirubin, *PLT* platelet, *ART* antiviral therapy; *p* < 0.05 was considered statistically significant. # indicates the factors were calculated by the Fisher exact test, otherwise they were calculated by Pearson’s χ2 test

### Development and internal assessment of a risk-scoring model in the derivation cohort

The 12 variables with a *p*-value of less than 0.1 in the univariate analysis were then entered into the multivariate logistic regression analysis. Among these variables, continuous variables were transformed into categorical variables, prior to multivariate logistic regression analysis. Table 2 shows the assignment of these variables. Multivariate analysis revealed age, stiff neck, impaired consciousness, blood urea, ICP, and CD4^+^ T-cell counts were potential risk factors for mortality in HIV/CM inpatients. A specific risk score (Table 3) assigned for each risk factor is derived by a calculation of each coefficient score (Table 3) multiplied by each point score (Table 2). Based on these six potential predictors and corresponding score component value, the risk prediction model was established. In other words, the clinical score of risk of mortality of individual HIV/CM patients was calculated using the following formula: Prognostic score = 2 × (CD4^+^ T-cell count) + 1 × (ICP) + 2.5 × (impaired consciousness) + 2 × (stiff neck) + 1 × (urea) + 1.5 × (age).

**Table 2:** Point scores of 386 HIV/CM inpatients in the derived cohort

	0	1	2	3
ICP (mmH_2_O)	< 200	200–250	251–350	> 350
CD4^+^ T-cell count (cells/μL)	> 50	≤50		
PLT (× 10^6^ /L)	> 60	≤60		
Age (years)	≤49	50–59	60–69	≥70
TBIL (μmol/L)	< 34	34–51	> 51	
Urea (mmol/L)	≤7.1	> 7.1		
HIV RNA (log10 copies/mL)	< 5	≥5		

*Abbreviations*: *ICP* intracranial pressure, *PLT* platelet, *TBIL* total bilirubin; *p* < 0.05 was considered statistically significant

**Table 3:** Independent predictors of mortality and corresponding score component value in HIV/CM individuals in the derived cohort

Predictors	Co-efficient	***p***-value	OR (95% CI)	Score of co-efficient	Score component value
Age (years)	1.110	<0.001	3.034(2.050–4.491)	1.5	
≤ 49					0
50–59					1.5
60–69					3
≥ 70					4.5
Impaired consciousness	1.841	<0.001	6.304(2.740–14.504)	2.5	
No					0
Yes					2.5
Stiff neck	1.502	0.001	4.492(1.844–10.944)	2	
No					0
Yes					2
ICP (mm H_2_O)	0.788	<0.001	2.200 (1.604–3.017)	1	
< 200					0
200–250					1
251–350					2
> 350					3
Urea (mmol/L)	0.904	0.040	2.470(1.041–5.864)	1	
≤ 7.1					0
> 7.1					1
CD4^+^ T-cell count (cells/μL)	1.505	0.002	4.503(1.756–11.547)	2	
> 50					0
≤ 50					2

*Abbreviations*: *ICP* intracranial pressure, *PLT* platelet, *TBIL* total bilirubin, *OR* odds ratio, *CI* confidence interval; *p* < 0.05 was considered statistically significant

**Fig. 2:**
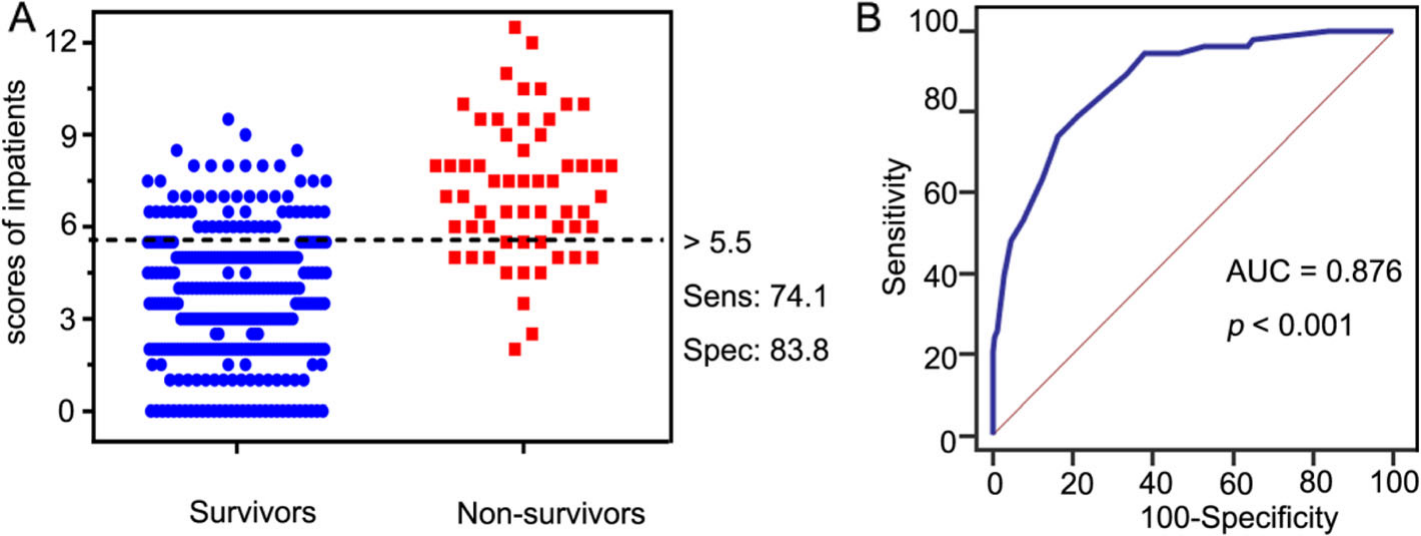
Mortality risk prediction rules for 386 HIV/CM inpatients in the derivation cohort. **A** Interactive dot diagram. The total score calculated by the prediction model labels the vertical axis of the above plot, and the clinical outcome of a patient during hospitalization marks the horizontal axis. The scores for each patient are displayed as dots on two vertical axes. The horizontal line indicates the cut-off point with the best separation. When using a score of 5.5 as the cut-off value to predict the mortality risk of HIV/CM inpatients, the sensitivity of our scoring system was 74.1% (95% CI, 0.610–0.847), and its specificity was 83.8% (95% CI,0.794–0.877). **B** The ROC curve. Each point on the ROC curve represents a sensitivity/specificity pair corresponding to a particular decision threshold. The AUC is a measure of how well a parameter can distinguish between two groups (survivors/non- survivors). ROC, Receiver operator characteristic; AUC, area under the ROC curve; Sens, sensitivity; Spec, specificity

Our screening score model showed good prediction performance (reflected in the Youden index, 57.98%), and had an AUC of 0.876 (95% CI, 0.838–0.907, *p* < 0.001) (Fig. 2). Using the ROC analysis, a total score greater than 5.5 was selected as the cut-off prediction value for high-risk patients. This cut-off point had a sensitivity of 74.1% (95% CI, 0.610–0.847) and specificity of 83.8% (95% CI, 0.794–0.877) at the maximum Youden index value. With this cut-off value, the positive predictive value (PPV) and negative predictive value (NPV) were 44.8% (95% CI, 0.378–0.520) and 94.8% (95% CI, 0.922–0.966), respectively.

To facilitate the clinical application of the predictive model, the study population with these risk factors was further divided into two subgroups (low-risk group, total score ≤ 5.5; high-risk group, total score > 5.5) according to their respective scores. The results indicated that patients with higher scores showed poorer prognosis in our cohort. In the derivation cohort, the cumulative mortality rate in the high-risk group was 44.8% (43/96), which was significantly higher than the mortality rate in the low-risk group (15/290, 5.2%) (*p* < 0.001) (Table 4).

**Table 4:** Predicted probability of mortality in HIV/CM inpatients in the derivation cohort and validation cohort

	Subgroups	Score	Survivors	Non-survivors	*p*
Derivation cohort	Low-risk group	≤5.5	275 (94.8%)	15 (5.2%)	< 0.001
	High-risk group	> 5.5	53 (55.2%)	43 (44.8%)	
Validation cohort	Low-risk group	≤5.5	125 (95.4%)	6 (4.6%)	< 0.001
	High-risk group	> 5.5	21 (55.3%)	17 (44.7%)	

**Fig. 3:**
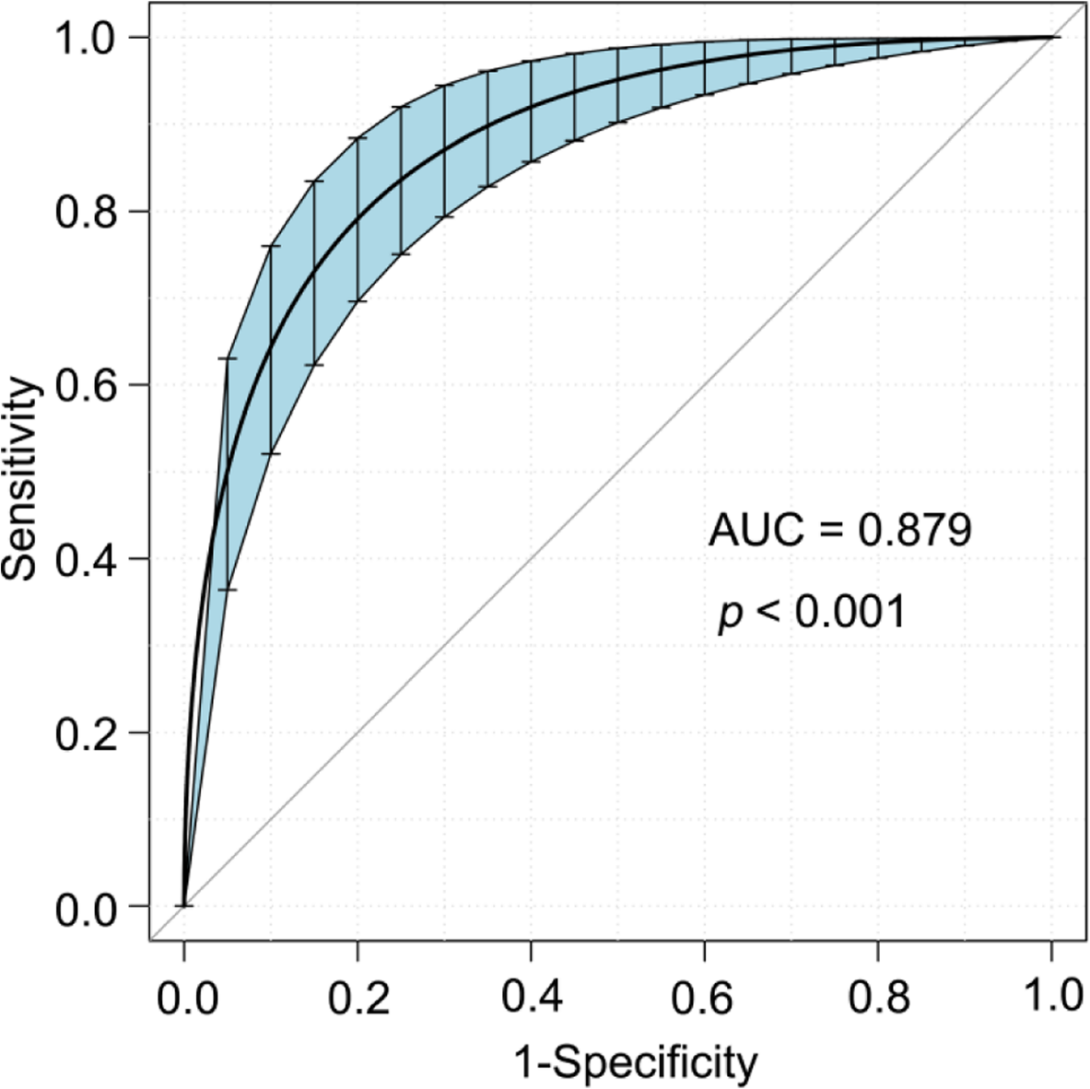
The receiver operating characteristic (ROC) curve for the internal validation of our scoring system using the bootstrap statistical method. AUC = area under the ROC curve

The AUC of the score for predicting mortality risk was 0.879 (95% CI, 0.829–0.921; Fig. 3) in the 1000 bootstrapped samples (internal validation). The sensitivity of our scoring system was 81.0% (95% CI, 0.682–0.897), its specificity was 81.7% (95% CI, 0.770–0.857), its PPV was 43.9% (95% CI, 0.344–0.538), its NPV was 96.1% (95% CI, 0.929–0.979). There was no significant difference between the observed and predicted mortality risk (Hosmer–Lemeshow goodness-of-fit test, *p* = 0.712), indicating that the model had a favorable goodness of fit.

### Discriminatory ability of the risk scoring model in the external validation cohort

**Fig. 4:**
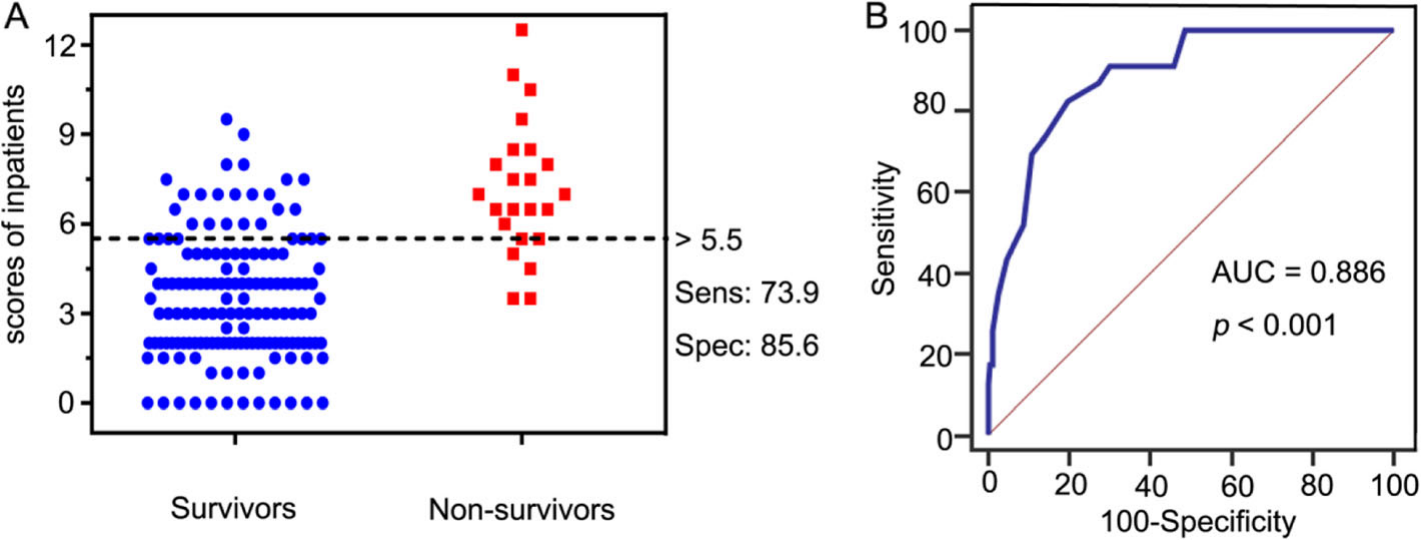
Mortality risk prediction rules for 169 HIV/CM inpatients in the validation cohort. **A** Interactive dot diagram. The total score calculated by the prediction model labels the vertical axis of the above plot, and the clinical outcome of a patient during hospitalization marks the horizontal axis. The scores for each patient are displayed as dots on two vertical axes. The horizontal line indicates the cut-off point with the best separation. When using a score of 5.5 as the cut-off value to predict the mortality risk of HIV/CM inpatients, the sensitivity of the risk scoring system was calculated to be 73.9% (95% CI, 0.516–0.898), and its specificity was 85.6% (95% CI, 0.789–0.909). **B** The ROC curve. Each point on the ROC curve represents a sensitivity/specificity pair corresponding to a particular decision threshold. The AUC is a measure of how well a parameter can distinguish between two groups (survivors/non- survivors). ROC, Receiver operator characteristic; AUC, area under the ROC curve; Sens, sensitivity; Spec, specificity

The performance of the new scoring model is externally verified by the validation cohort. The comparison results from the validation cohort showed that the model had good performance in predicting mortality (AUC = 0.886, 95% CI 0.838–0.907, Fig. 4), and its goodness of fit was satisfactory (*p* = 0.152). Sensitivity of the risk scoring model was calculated to be 73.9% (95% CI, 0.516–0.898), specificity was 85.6% (95% CI, 0.789–0.909), PPV was 44.7% (95% CI, 0.337–0.563), NPV was 95.4% (95% CI, 0.913–0.977), and the Youden index was 62.8%. This indicates that the predictive model is effective in predicting mortality in HIV/CM inpatients. Table 4 indicates that there was a significant difference in the cumulative mortality in the validation cohort between lower and higher predictive risk group of HIV/CM patients (*p* < 0.001) (6/131, 4.6% &17/38, 44.7%).

## Discussion

As is well recognized, CM is one of the most prevalent and potentially lethal opportunistic infectious diseases affecting HIV positive patients, and causes approximately 181,000 deaths per year worldwide [17]. All-cause mortality during the 28 days after diagnosis of CM was 15.0% in our study, which is concernedly high, although lower than that observed in other studies [18–20]. It is therefore vital for clinicians to determine the risk factors that favor the development of HIV/CM. Although many past studies have investigated potential prognostic factors for CM, consensus regarding prognostic risk factors remains elusive due to utilization of different therapeutic regimens in different studies or different regions.

In the present study, we developed and validated a risk scoring model for HIV/CM using the data of 555 subjects afflicted with HIV-associated CM. After verifying the scoring model, the following six risk predictors were selected: age, stiff neck, impaired consciousness, blood urea, ICP, and CD4^+^ T-cell counts of HIV/CM patients at admission. Based on these risk factors, a novel model for HIV/CM prognostication was built, which, to the best of our knowledge, is the first prognostic assessment model for use in patients with HIV/CM.

Compared to several previous studies which have also identified some similar risk factors associated with poor prognosis in CM patients [9, 21–25], the present study differs in the following aspects: 1) All of our study participants were HIV-infected persons while the study participants in the majority of previous studies were HIV-negative persons, and the total number of participants in this study was 555, which is a relatively large sample size with regards to these types of studies. 2) Some studies chose neurocognitive impairment, seizures, neurological sequelae, relapse, or treatment effectiveness as outcome indicators in their analysis of risk factors for HIV/CM, rather than death as an outcome. 3) Our study established a convenient and novel predictive model based on the previously mentioned risk factors, and the model was then further validated using data from another 169 HIV/CM patients.

In our study, mortality was higher for patients older than 50 years of age, and risk scores increased by as much as 1.5 points for each additional 10 years of age. A recent study found that 8-week mortality for patients who were ≥ 50 years was higher than those who were < 50 years (28.6% vs 17.3%) [26]. In a retrospective analysis, older age (> 50 years) was independently associated with 2-week mortality of patients with HIV complicated with CM [8]. Moreover, patients aged ≥50 years were almost 4 times more likely to die than those aged < 50 years [8]. Qu et al. reported that the survival rate of patients with CM aged over 60 years was worse than that of those younger than 60 years of age [27]. These findings indicate that advanced age (≥50 years old) is an important predictor of mortality risk in patients with CM. Globally, the life span of people living with HIV is now much longer than in the past, suggesting that clinicians need to focus on the age of patients, pay appropriate attention to the patient’s clinical condition, and make appropriate therapeutic adjustments timeously.

Elevated ICP is common during the diagnostic workup for CM, with more than half of HIV/CM patients reportedly having an opening ICP of over 250 mmH_2_O, and more than a quarter having an opening ICP of above 350 mmH_2_O [28]. Raised ICP at baseline diagnosis of CM (≥250 mmH_2_O) is associated with altered mental status, visual and hearing loss, other symptoms of cranial nerve dysfunction, and even impaired consciousness or death. In a large trial of CM treatment, almost all the early deaths (13/14) and 40% of the deaths during weeks 3 through week 10 were associated with elevated ICP (>250 mmH_2_O) [29]. Graybill and colleagues also found that short-term survival of HIV/CM patients was significantly lower in the 250–349 mmH_2_O pressure group and > 350 mmH_2_O pressure group, than those with pretreatment opening pressures <250 mmH_2_O (according to Kaplan-Meier analysis) [30]. Another observational retrospective study also found that HIV/CM patients in the high ICP group (> 250 mmH_2_O) had a higher mortality rate (21/57, 36.8%) than those who had a normal ICP at baseline (6/23, 26.1%) [31]. Consistent with the findings of these earlier trials, in our study, elevated ICP did negatively impact on outcomes at 4 weeks. Stepwise multivariate analysis indicated that as ICP increased, the risk of death for HIV-infected patients with CM also increased. Aggressive management of increased ICP is therefore suggested in the treatment of CM, with the goal of achieving an ICP of 200 H_2_O or less, or 50% of the initial opening pressure measured [32]. At the same time, current guidelines call for subsequent appropriate drug treatment of CM to maintain normal ICP to control symptoms of intracranial hypertension [33]. Of note, CSF opening pressures should be measured as an integral part of the initial evaluation. If the initial ICP is normal, the ICP should be further measured at least once more. For patients with elevated baseline ICP (> 250 mmH_2_O), additional LP (including daily LP) may be performed as necessary until the pressure is adequately controlled [34]. In a study of 26 patients with CM, 7 of 14 patients who did not have their initial ICP measured developed neuropathies during therapy, compared to only 1 of 12 patients who underwent ICP monitoring [35]. This suggests that deviations from standard ICP management could likely lead to poor neurological outcomes. As such, these analyses support the call for continued vigilance regarding ICP management during the window of opportunity shortly after diagnosis of HIV/CM, including timely monitoring of CSF pressure and effective control of ICP during antifungal therapy.

Low CD4^+^ T-cell count is associated with a poor prognosis of CM. Pastick’s study found that a CD4^+^ T-cell count of < 50 cells/μL was an important predictor of epilepsy in patients with HIV/CM [9]. A study in Uganda indicated that a CD4^+^ T-cell count of < 50 cells/μL was associated with higher 18-week mortality, compared to a CD4^+^ T-cell count of 50–100 cells/μL and 100–200 cells/μL [36]. This finding was similar to our observation in the present study, suggesting that monitoring of CD4^+^ T-cell levels in HIV/CM helps identify patients with increased risk of poor prognosis. Impaired consciousness and stiff neck are common clinical manifestations and symptoms in patients with CM, and their incidence was significantly higher in patients with unfavorable outcomes [25]. Wu et al. reported that the presence of impaired consciousness increased the risk of poor prognosis by a factor of 4.4 [24]. High urea nitrogen level is often a manifestation of abnormal renal function. Our results in this study found that high urea nitrogen was an important risk factor for mortality in HIV/CM patients, which indicates that periodic screening for, and timely normalization of urea nitrogen were necessary in patients with CM.

In summary, our novel scoring model is likely to be useful for predicting outcomes in patients with HIV/CM. Utilizing our scoring model, clinicians may stratify affected patients so that they may decide upon appropriate patient-specific treatment regimens, or, if indicated, make early referrals to specialist care, particularly in primary care settings. Higher scores obtained via our novel system may serve as an early warning for this particular group of patients. Low scores may indicate that affected patients may be placed in a milder risk category, which may help reduce inappropriate utilization and wastage of expensive medical resources [37]. Additionally, the listed predictive risk factors in our study would be paid additional attention to by attending physicians along the entire course of the patient’s illness, as positive changes to the risk factors during the illness course, may positively influence HIV/CM outcomes. It is hoped that our novel scoring model may be incorporated into the diagnostic and therapeutic workup for newly-diagnosed HIV/CM patients so as to improve outcomes in this patient population.

The present study has limitations. First, as our study was retrospective in nature, it therefore may be susceptible to the various limitations inherent to retrospective studies in general. Second, the participants in our study were all Chinese nationals, and whether our proposed scoring model can successfully be used for other population or racial groups is unknown, and warrants further research. Additionally, there are a few potential risk factors for which we did not have adequate information in our data set, and were not included in our data analysis, and inclusion of these risk factors into our model may further improve the prognostic accuracy of our mortality risk score. These potential risk factors include cryptococcal antigen titers (CrAg titers) in both serum and CSF, body mass index (BMI), CSF protein levels, CSF chloride levels, dose and course length for Amphotericin B, and other central nervous system symptoms (e.g., vision loss, hearing loss, etc.). There is evidence that the higher CrAg titers in both serum and CSF are associated with an increased risk of mortality in HIV/CM [38], and thus its inclusion as a potential predictive risk factor in the development of future risk scores for HIV/CM patients should be considered. However, hospitals and clinics that do not have the capacity to perform these additional tests may safely use our novel scoring model in order to accurately stratify risk in HIV patients with CM.

## Conclusions

In conclusion, our study outlines a simple and useful scoring model developed to predict mortality in HIV/CM. The sensitivity, specificity, and accuracy of the scoring system after verification are meaningful and applicable to clinical medical practice in areas where HIV/CM prevalence and mortality remains notably high. We envision that our scoring model may be widely used in these settings in order to effect demonstrable reductions in HIV/CM mortality.

## Data Availability

The datasets analyzed during the present study are available from the corresponding author upon reasonable request.

## Abbreviations

CM: Cryptococcal meningitis
PLT: Platelet
ROC: Receiver operating characteristic
TBIL: Total bilirubin
HGB: Hemoglobin
AmB: Amphotericin B
ART: Antiviral therapy
OR: Odds ratios
95% CI: 95% confidence interval
HIV: Human Immunodeficiency Virus
WHO: World Health Organization
SPSS: Statistical Package for the Social Sciences
